# Evidence of effective scrapie transmission via colostrum and milk in sheep

**DOI:** 10.1186/1746-6148-9-99

**Published:** 2013-05-07

**Authors:** Timm Konold, S Jo Moore, Susan J Bellworthy, Linda A Terry, Leigh Thorne, Andrew Ramsay, F Javier Salguero, Marion M Simmons, Hugh A Simmons

**Affiliations:** 1 Specialist Scientific Support Department, Animal Health and Veterinary Laboratories Agency Weybridge, New Haw Addlestone, Surrey KT15 3NB, UK; 2 Formerly – Veterinary Laboratories Agency Weybridge, New Haw, Addlestone, Surrey KT15 3NB, UK; 3 School of Veterinary and Biomedical Sciences, Murdoch University, South Street, Murdoch, WA 6150, Australia; 4 TSE Department, Animal Health and Veterinary Laboratories Agency, New Haw, Addlestone, Surrey KT15 3NB, UK

**Keywords:** Transmissible spongiform encephalopathy, Scrapie, Sheep, Milk, Colostrum, Transmission, PMCA, Prion protein, RAMALT, Copper

## Abstract

**Background:**

Evidence for scrapie transmission from *VRQ/VRQ* ewes to lambs via milk was first reported in 2008 but in that study there were concerns that lateral transmission may have contributed to the high transmission rate observed since five control lambs housed with the milk recipients also became infected. This report provides further information obtained from two follow-up studies, one where milk recipients were housed separately after milk consumption to confirm the validity of the high scrapie transmission rate via milk and the second to assess any difference in infectivity from colostrum and subsequent milk. Protein misfolding cyclic amplification (PMCA) was also used to detect prion protein in milk samples as a comparison with the infectivity data and extended to milk samples from ewes without a *VRQ* allele.

**Results:**

Seven pairs of lambs fed colostrum and milk individually from seven scrapie-affected sheep (pre-clinical or clinical) presented with disease-associated prion protein, PrP^d^, in rectal lymphoid tissue at 4–5 months of age. Five further pairs of lambs fed either colostrum or subsequent milk from five pre-clinical scrapie-affected sheep equally presented with PrP^d^ in lymphoid tissue by 9 months of age. Nine sheep were lost due to intercurrent diseases but all remaining milk or colostrum recipients, including those in the original study with the lateral transmission controls, developed clinical signs of scrapie from 19 months of age and scrapie was confirmed by brain examination. Unexposed control sheep totalling 19 across all three studies showed no evidence of infection.

Scrapie PrP was amplified repeatedly by PMCA in all tested milk samples from scrapie-affected *VRQ/VRQ* sheep, and in one scrapie-affected *ARQ/ARQ* sheep. By contrast, milk samples from five *VRQ/VRQ* and 11 *ARQ/ARQ* scrapie-free sheep did not have detectable scrapie PrP on repeated tests.

**Conclusions:**

Feeding of milk from scrapie-affected sheep results in a high transmission rate in *VRQ/VRQ* sheep and both colostrum and milk transmit scrapie. Detection of scrapie prion protein in individual milk samples from scrapie-affected ewes confirms PMCA as a valuable *in vitro* test.

## Background

Until recently the main source of transmission of classical scrapie, a transmissible spongiform encephalopathy (TSE) of sheep, was believed to be the placenta [1]. We have previously shown that colostrum and milk collected from scrapie-affected ewes was able to transmit disease when fed to lambs although we were unable to determine whether colostrum or milk alone was infectious and whether lateral transmission between milk recipient lambs may have contributed to the apparently high transmission rate because milk recipient sheep were mixed after all milk had been consumed [2]. This previously reported study (Study 1) utilised Cheviot sheep with a prion protein genotype *VV*_*136*_*RR*_*154*_*QQ*_*171*_, which in this breed is associated with high scrapie susceptibility [3] and results in accumulation of immunohistochemically detectable disease-associated prion protein (PrP^d^) in the lymphoreticular system (LRS) at a young age [4]. A subsequent study in transgenic mice over-expressing ovine *VRQ* prion protein (Tg338 mice) demonstrated that both colostrum and milk from *VRQ/VRQ* and *ARQ/VRQ* sheep, after concentrating scrapie prion protein on magnetic beads, were able to transmit scrapie [5]. In a separate experiment it was shown that milk from *ARQ/ARQ* sheep orally inoculated with scrapie brain homogenate transmits scrapie to lambs [6]. Both studies used milk from sheep co-infected with the maedi-visna virus (MVV), which can cause lymphofollicular mastitis that may contribute to prion secretion into milk [7] compared to a healthy udder. Others used a sensitive prion detection method, protein misfolding cyclic amplification (PMCA), to test milk from scrapie-exposed sheep and found the presence of scrapie prion protein (PrP^sc^) in milk of sheep carrying at least one *VRQ* allele [8] although it is not known whether this would lead to disease transmission in a sheep because of the sometimes poor association between PrP^sc^ detection and infectivity [9,10].

The present work was undertaken following the results from the initial study (Study 1), updated here, to further study scrapie transmission via milk by using the natural host, namely to confirm the high scrapie transmission rate via milk whilst lateral transmission was prevented by housing lambs separately after milk consumption (Study 2) and to investigate whether colostrum or subsequent milk in a lactation are equally effective in the transmission of scrapie in sheep (Study 3). In a parallel study, milk samples from sheep, which included milk from some sheep used for the transmission study, were examined for presence of PrP^sc^ using PMCA to allow comparison with the *in vivo* studies.

## Results

### Study 1. Feeding milk from scrapie infected sheep to lambs with lambs housed together

#### Recipient lambs and lateral transmission controls

**Figure 1:**
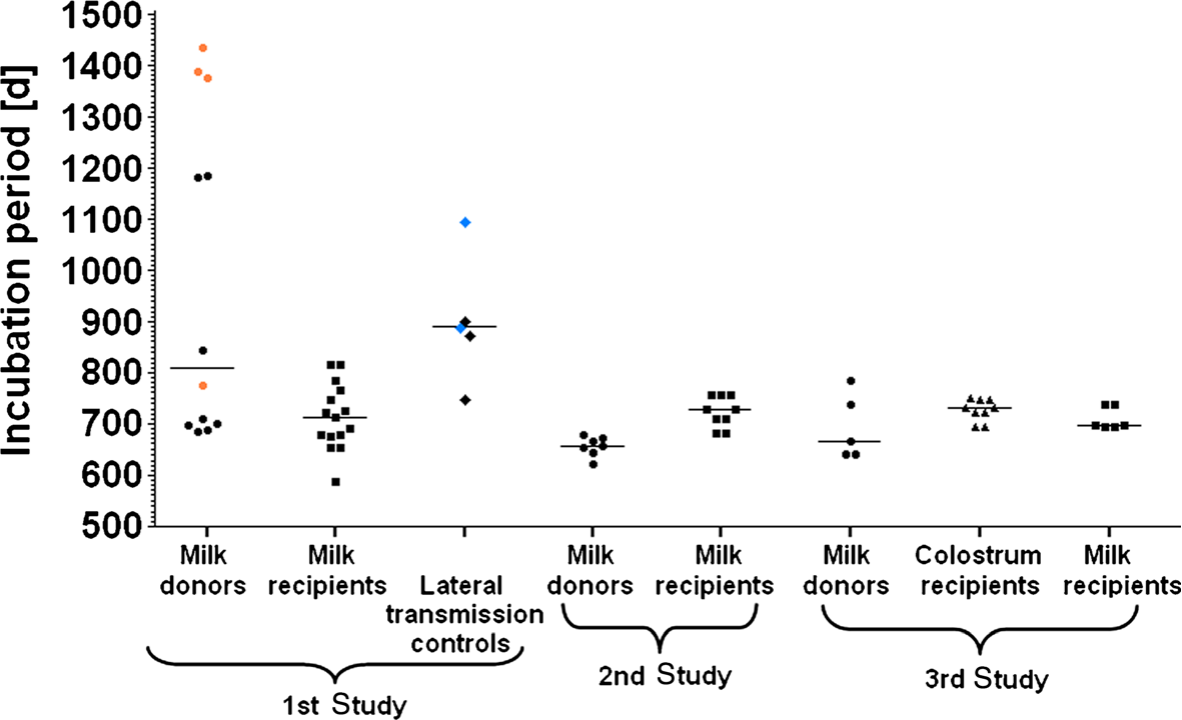
**Incubation periods in days of scrapie milk donor, milk recipient sheep and lateral transmission controls.** The median incubation periods for milk donors were 808.5 (range: 683–1436) days in Study 1, 657 (623–679) days in Study 2 and 666 (639–784) days in Study 3. The median incubation periods for milk recipients were 713 (588–816) days in Study 1, 729 (682–755) in Study 2 and 696.5 (694–738) days in Study 3. Lateral transmission controls in Study 1 had a median incubation period of 889 (range: 746–1095) days and colostrum recipients in Study 3 730 (693–749) days.

All of the 15 remaining scrapie milk recipients (out of a total of 18 sheep, see “Additional file 1: summary” for an overview and [2]) developed clinical signs of scrapie and were culled at 19–27 months of age (see “Additional file 2: 07-1092” showing a scrapie milk recipient at clinical end-point) whereas the five lateral transmission controls were culled with signs of scrapie at 27–40 months of age (25–36 months post exposure, see “Additional file 3: 07-1246” showing a lateral transmission control at end-stage disease). Immunohistochemical and Western blot examination of the brains confirmed the clinical diagnosis in all sheep (presence of PrP^d^ or its proteinase-resistant form, PrP^res^). All except for the last culled lateral transmission control, which displayed alopecia with skin lesions and a positive scratch test at the time of cull, presented with vacuolar changes in the obex. This sheep was also the only one that had no detectable PrP^d^ in recto-anal mucosa-associated lymphoid tissue (RAMALT) at 14.5 months post exposure although it was present in a further biopsy at 34 months of age. The incubation periods of the scrapie-affected milk recipients (= time from birth to cull), lateral transmission controls (= time from exposure (mixing) to cull) and those of the milk donor sheep are shown in Figure 1.

#### Building controls

One building control was lost due to acquired thoracic vertebral malformation at 14 months of age. The remaining eight controls were culled at 39 months of age, one of which displayed a positive scratch test inconsistently prior to cull although there was no evidence of pruritic behaviour. All sheep were negative for scrapie by immunohistochemical examination of obex, distal ileum, mesenteric lymph node and spleen as well as Western blot examination of the caudal medulla.

An overview of the experimental outcome in this study is presented in “Additional file 1: summary”.

### Study 2. Feeding milk from scrapie infected sheep to lambs without housing lambs together

#### Donor sheep

None of the scrapie-affected ewes that provided the milk had any evidence of mastitis based on inspection of udder and milk. Lactation onset, duration, clinical onset with respect to milk collection, weekly somatic cell count (SCC) and age at cull for each milk donor ewe are displayed in Table 1. Immunohistochemical examination revealed PrP^d^ in the inguinal lymph node of all ewes but not in the involuted mammary gland.

**Table 1:** Details of sheep that provided milk to lambs without mixing of lambs (Study 2)

**Donor ewe**	**Start of lactation**	**Days of lactation**	**Cull with strong signs of scrapie**	**Unequivocal scrapie signs observed**	**Somatic cell count (weekly samples)**	**Milk provided per recipient**
06-1058	18m 14d	68	22m 10d	Lactation day 24	49;45;42;22;39;62;27;49;49	33
06-1404	18m 10d	70	21m 13d	Lactation day 25	153;97;178;179;22;68;20;1318;1304*	50
06-1433	18m 11d	58	21m 25d	30 d after lactation end	30;161;1630**;63;40;84;21;229	27.5
06-1501	18m 11d	55	21m 17d	30 d after lactation end	19;81;483;59;24;109;12;161	16.5
06-1510	18m 7d	60	20m 14d	2 d after lactation end	39;46;60;11;89;56;16;68	24.5
06-1514	18m 9d	64	22m 2d	None, culled with mild signs	30;45;58;51;18;27;11;31;185	23.5
06-1520	18m 8d	58	21m 3d	Lactation day 23	26;67;34;23;18;41;31;108	18.5

All ewes were scrapie-positive based on brain examination (vacuolar changes and presence of PrP^d^/PrP^res^). The age is displayed in months (m) and days (d), with months displayed in calendar months; the somatic cell count is displayed in 10^3^ cells/ml; milk volume is displayed in litres (rounded down).

* *Staphylococcus epidermidis* isolated.

** No bacteria isolated.

#### Recipient lambs

RAMALT biopsies of the 14 lambs were taken at 136–138 days of age. This was repeated in three lambs that had less than four follicles in the section examined. It was considered that insufficient tissue had been taken to reach a diagnosis. The results revealed that at least one lamb of each pair was scrapie-positive (PrP^d^ accumulation in lymphoid follicles). Five lambs subsequently developed sudden dullness with jaundice and were culled at 183 (lamb 08–1351, milk donor 06–1501), 190 (lambs 08–1343 and 08–1344, milk donor 06–1514), 205 and 210 days of age (lamb 08–1346 and lamb 08–1345 respectively, milk donor 06–1404). Abundant copper staining in the livers of these lambs using special stains (rhodanine and rubeanic acid) together with liver copper concentrations of 15.8-22.2 mmol/kg compared to normal values of ≤7.85 mmol/kg [11] were indicative of chronic copper intoxication. The cause could not be determined: copper levels in selected samples (drinking water, food, bedding) ranged from 0.01 to 9.16 mg/kg, concentrated food also contained the copper-antagonist molybdenum, and other sheep on the premises, which were kept in a different building but fed the same diet, did not develop disease. As a consequence, a 3-month course of 0.1 g ammonium molybdate and 1 g sodium sulphate dissolved in 10 ml of water administered daily *per os* was initiated as prophylactic treatment to enhance excretion of copper and no further cases were observed. All five lambs presented with PrP^d^ accumulation in distal ileum, spleen (except for 08–1351) and mesenteric lymph node but not in the brain. All remaining scrapie milk recipients developed clinical signs of scrapie resulting in their cull at 22–25 months of age. Scrapie was confirmed by histopathological (presence of vacuolar changes), immunohistochemical (presence of PrP^d^) and Western blot examination (presence of PrP^res^) of the brains.

The incubation periods of the scrapie-affected milk recipient sheep and the milk donor sheep are displayed in Figure 1.

#### Building controls

RAMALT biopsies taken around the same time from three of the five building controls did not show detectable PrP^d^ in the sampled section; the other two sheep were re-sampled 141 days later due to insufficient tissue being collected but only one had enough tissue for a diagnosis, which was scrapie-negative (absence of PrP^d^ in the section). No further biopsies were taken.

Controls were culled with no evidence of clinical disease at 25 months of age, after the last scrapie milk recipient had been culled and were negative for scrapie by postmortem tests.

See “Additional file 1: summary”, which provides an overview of the experimental outcome in this study.

### Study 3. Feeding colostrum and milk from scrapie infected sheep to separate lambs

#### Donor sheep

None of the scrapie-affected ewes that provided the milk had any evidence of mastitis based on inspection of udder and milk. Breed, lactation onset, duration, clinical onset with respect to milk collection, weekly SCC and age at cull for each milk donor ewe are displayed in Table 2. See also Additional file 4: 07–1288 showing the clinical presentation of this sheep at milking and prior to cull, several months after the milk was collected. All ewes presented with detectable PrP^d^ in inguinal lymph node but not in the involuted mammary gland. The incubation periods of the donor sheep are shown in Figure 1.

**Table 2:** Details of sheep that provided colostrum and milk to separately fed lambs (Study 3)

**Donor ewe**	**Breed**	**Start of lactation**	**Days of lactation**	**Cull**	**Unequivocal or strong scrapie signs observed**	**Somatic cell count (weekly samples)**	**Colostrum provided per recipient**	**Milk provided per recipient**
07-1006	PD	21m 2d	42	24m 6d	48 d after end of lactation*	57;41;56;32;8;11	1	11
07-1287	PD x Fries	17m 25d	54	21m 25d	35 d after end of lactation	42;47;27;9;17;7;1;6	1.5	19.5
07-1288	PD x Fries	17m 24d	54	20m 29d	35 d after end of lactation*	18;12;34;26	1.5	16.5
07-1292	PD x Fries	17m 25d	52	20m 29d	37 d after end of lactation*	31;26;28;35;8;15;25	1.5	21
07-1337	PD x Fries	17m 21d	46	25m 23d	45 d after end of lactation	14;10;27;10;46;32	2	11.5

All ewes were scrapie-positive based on brain examination (vacuolar changes and presence of PrP^d^/PrP^res^). The age is displayed in months (m) and days (d), with months displayed in calendar months; the somatic cell count is displayed in 10^3^ cells/ml; volume of colostrum or milk is displayed in litres (rounded down).

PD, Poll Dorset, *Fries*, Friesland.

* Sheep displayed strong scrapie signs.

#### Milk recipient lambs

**Figure 2:**
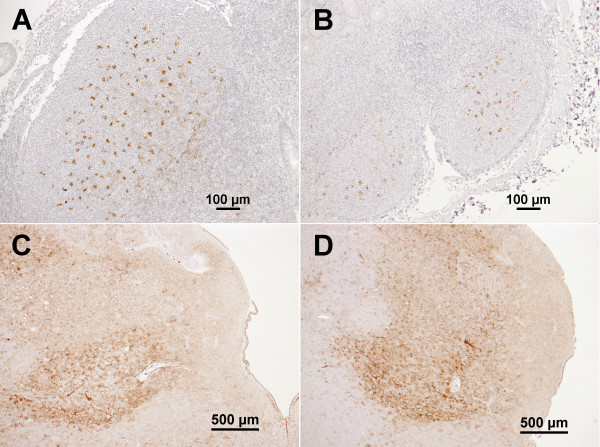
**PrP**^**d **^**accumulation in colostrum recipient 09–1447 and milk recipient 09–1426 in Study 3 (donor: 07–1288).** Immunolabelling with antibody R145. **A**) RAMALT of 09–1447 at 5 months of age. **B**) RAMALT of 09–1426 at 5 months of age. **C**) Obex of 09–1447, culled at 24 months of age. **D**) Obex of 09–1426, culled at 24 months of age. PrP^d^ immunolabelling is evident in the lymphoid follicles of the rectal mucosa, which is suggestive of infection with the scrapie agent following ingestion of colostrum (**A**) and milk (**B**), from scrapie sheep 07–1288. After cull with clinical signs of scrapie, both sheep presented with PrP^d^ immunolabelling in the brain [here: parasympathetic nucleus of the vagus nerve: **C** (colostrum recipient) and **D** (milk recipient)] consistent with the diagnosis of scrapie.

One scrapie-milk challenged lamb (09–1436, milk donor: 07–1287) developed anaemia and ill-thrift and was culled at 44 days of age. This lamb presented with PrP^d^ in lymphoid follicles of the distal ileum indicative of early pre-clinical infection (with no detectable PrP^d^ in spleen and mesenteric lymph node). RAMALT biopsies of the remaining lambs (five pairs fed colostrum from five scrapie-affected ewes, four pairs and a single lamb fed milk from the same ewes) were taken at 138–141 days of age and repeated for three lambs with less than five follicles in the section examined. At least one lamb of each scrapie colostrum-fed pair and at least one lamb of four of five scrapie milk-fed pairs (including the single lamb that was the companion of the culled lamb) were scrapie-positive (PrP^d^ accumulation in lymphoid follicles, see Figure 2A and B as example). At 278 days of age, further RAMALT biopsies were taken from the scrapie milk-fed pair that had previously been scrapie-negative: PrP^d^ was found in RAMALT of both sheep.

Similar to Study 2, three sheep developed jaundice and dullness and were culled: the former companion of the anaemic lamb at 179 days of age (09–1438), a scrapie milk recipient (09–1435, milk donor: 07–1337) at 186 days and a scrapie colostrum recipient (09–1465, colostrum donor: 07–1292) at 368 days of age. Detectable PrP^d^ was found in distal ileum and mesenteric lymph node (all three), spleen (two: 09–1438, 09–1465) and obex (one: 09–1465, immunolabelling restricted to the parasympathetic nucleus of the vagus nerve). All had postmortem findings consistent with chronic copper intoxication (liver copper 10.4-15.6 mmol/kg), also resulting in hepatic encephalopathy in colostrum recipient 09–1465, which presented with severe white matter vacuolation in the obex, particularly in the reticular formation. The source of the intoxication could again not be determined. All remaining sheep were treated orally against copper intoxication as above for 33 days after the first occurrence of copper intoxication and for 67 days following the second occurrence.

A further sheep (09–1432, milk donor 07–1332) was culled at 19 months of age because of malnutrition following the development of marked maxillary prognathism. A detailed clinical examination was not carried out prior to cull but vacuolar changes and PrP^d^ accumulation were detected in the brain histopathologically, and Western blot examination of the brain revealed PrP^res^.

The remaining 15 sheep (nine colostrum recipients, six milk recipients) were culled with clinical signs of scrapie at 23–25 months of age. All presented with vacuolar changes and PrP^d^ accumulation in the obex (see Figure 2C and D as examples of the same sheep that previously presented with PrP^d^ in RAMALT) and PrP^res^ was demonstrated by Western blot examination of a sample of the caudal medulla.

#### Building controls

RAMALT biopsies taken around the same time from the four building controls did not have detectable PrP^d^ in the section. No further biopsies were taken. All sheep were clinically healthy when culled at 25 months of age and free from scrapie based on postmortem tests.

An overview of the colostrum and milk donors and the corresponding recipients with the experimental outcome is provided in “Additional file 1: summary”.

### Application of PMCA to detect PrP^sc^ in milk

Detection of PrP^sc^ was achieved after serial PMCA from all 25 milk samples taken from the six *VRQ/VRQ* scrapie-affected sheep (Table 3; 96% positive tests, 4 replicates for each sample). The day of lactation did not appear to influence the outcome of the test indicating that scrapie prion protein was present throughout lactation. PrP^sc^ was amplified from one milk sample from a scrapie-infected ewe that did not transmit scrapie to a lamb. Positive results were also observed in amplified samples from three tests of a total of 80 tests performed on 20 control milk samples (3.8%) from five scrapie-free *VRQ/VRQ* sheep. These three positive results were not obtained from the same sample or in the same assay and are unlikely to represent infectivity and possible explanations are discussed below.

**Table 3:** **Details of *VRQ/VRQ *ewes that provided milk for PMCA**

**Milk donor**	**Infection status**	**Scrapie transmission to lambs**	**Lactation day**	**Number of positive tests of a total of four tests**
07-1006	Scrapie	Yes	7	**4**
(Study 3)			13	**4**
			42	**4**
07-1337	Scrapie	Yes	8	**4**
(Study 3)			14	**4**
			28	**4**
			35	**4**
			43	**4**
07-1288	Scrapie	Yes	7	**4**
(Study 3)			13	**4**
			20	**4**
			27	**4**
			42	**4**
			48	**4**
07-1287	Scrapie	Yes	6	**4**
(Study 3)			19	**4**
			26	**4**
			41	**3**
			47	**4**
07-1292	Scrapie	Yes	6	**2**
(Study 3)			19	**4**
			26	**4**
			41	**3**
			47	**4**
139/6	Scrapie	No*	12	**4**
(Study 1)				
M340	Scrapie-free	Not tested	7	0
			14	0
			21	0
			28	0
N290	Scrapie-free	Not tested	10	**1**
			17	0
			24	0
			31	0
N531	Scrapie-free	Not tested	8	0
			15	**1**
			22	**1**
			29	0
P201	Scrapie-free	Not tested	9	0
			16	0
			23	0
			30	0
P381	Scrapie-free	Not tested	10	0
			17	0
			24	0
			31	0

* Culled at 105 days of age.

Positive samples are in bold font.

All scrapie-free sheep were Cheviot ewes, sheep 139/6 was a Poll Dorset × Friesland, see Table 2 for breeds of the other scrapie-affected sheep.

Furthermore milk from three *A*_136_*R*_154_ homozygous sheep carrying at least one *Q*_*171*_ allele (abbreviated to *ARX/ARQ*) and infected with either BSE or scrapie were tested for PrP^sc^ by PMCA. Only the milk from one *ARQ/ARQ* scrapie field case (134/11) produced a positive result from the colostrum sample from each udder half. As observed for the *VRQ* controls, two of the 11 milk samples (total 44 tests, 4.5%) from the *ARQ/ARQ* control sheep also yielded a positive result (see Table 4).

**Table 4:** **Details of *ARQ/ARX *ewes that provided milk for PMCA**

**Milk donor**	**Infection status**	**Lactation day**	**Result (positive out of 4 tests)**
06-1625	BSE	50	0 (L)
			0 (R)
127/11	Scrapie	2	0 (L)
			0 (R)
134/11	Scrapie	Unknown	**4** (L)
		(colostrum-like)	**4** (R)
K193	Scrapie-free	19	0
L90	Scrapie-free	23	0
M365	Scrapie-free	26	**1**
M395	Scrapie-free	21	0
M405	Scrapie-free	26	0
M447	Scrapie-free	24	**1**
M448	Scrapie-free	24	0
M533	Scrapie-free	23	0
N548	Scrapie-free	22	0
P181	Scrapie-free	23	0
P454	Scrapie-free	25	0

Positive samples are in bold font.

All scrapie-free sheep and the BSE-affected sheep were Suffolk, both scrapie field cases were 24 month-old Mule crossbreeds. L and R refer to the left and right side of the udder where milk was collected from.

PrP^d^ was detected immunohistochemically in the inguinal lymph nodes of the BSE-infected sheep and scrapie field case 134/11 but not in the lymph node of scrapie field case 127/11 or any mammary gland.

## Discussion

A recent epidemiological study has found an increased incidence of scrapie in the offspring of scrapie-affected ewes, even after controlling for the confounding effect of PrP genotype, which is suggestive of maternal transmission [12]. The authors further suggested that if the transmission occurs postnatally it is likely to occur rapidly after birth since early removal and artificial raising of lambs reduced the scrapie incidence only if the dams within a flock with scrapie were healthy and not yet themselves affected by scrapie [13]. The present studies (Studies 2 and 3) have demonstrated that milk, including colostrum, would cause this scrapie infection in *VRQ/VRQ* lambs. Although mixing of milk recipients after milk consumption in Study 1 meant lateral infection could not be ruled out as contributing to a high transmission rate [2], the transmission rate remained 100% in Study 2 where milk recipients were not mixed after milk consumption confirming that milk is a highly effective vehicle of scrapie transmission. Feeding milk or colostrum from scrapie infected sheep separately to different lambs all within 24 hours after birth (Study 3) was shown to be infectious to all lambs, even if as little as 1.5 litres of colostrum was fed. It is not known whether an equal volume of milk would have been sufficient to transmit disease. A similar volume of milk from pre-clinical scrapie-affected *ARQ/ARQ* sheep was able to infect *ARQ/ARQ* lambs [6], but transmission was only achieved with milk from sheep co-infected with MVV, which produced chronic lymphofollicular mastitis, and not with milk from scrapie-only affected sheep.

Since its first publication in 2001 [14] PMCA has become a widely used technique to achieve PrP^sc^ detection in fluids from scrapie-affected sheep, which contain amounts of PrP^sc^ that were previously undetectable by other tests [8,15-17]. This technique is based on the phenomenon that minute amounts of PrP^sc^ in the sample are able to convert cellular prion protein, which is added as substrate, into the disease-associated form and – by using serial cycles of incubation and sonication – is able to amplify PrP^sc^ in quantities that are detectable by rapid postmortem tests. However, on rare occasions prion protein was also detected in samples from non-TSE affected animals, and the product was indistinguishable from PrP^sc^. It was hypothesised that this was either the result of *de novo* generation of PrP^sc^ or due to cross-contamination of samples [18,19]. In the current study, PrP^sc^ was detected in milk from control sheep, which belonged to a flock that is free from classical scrapie and were thus extremely unlikely to shed prions via milk. In our experimental system it is not known whether these apparently spurious positive data result from contamination or the generation of new PrP^sc^. The inclusion of polyA in the reaction renders this methods ultra sensitive and we cannot preclude that extremely small amounts of contamination give rise to these positive samples. Nonetheless, the observation that 96% of tests were positive from sheep with milk known to be infectious to lambs is supportive of an association with infectivity. These results were in good agreement with those from the transmission study. One exception was the milk from a single scrapie-affected ewe in Study 1 (milk recipients were mixed after milk consumption), where PMCA analysis produced a consistently positive result (four of four tests) in the sample tested, but there was no evidence of scrapie transmission to the recipient [2]. Although PrP^d^ was not found in any of the tissues examined at necropsy when it was culled for other reasons at 105 days of age, it may have been too early in the incubation period for PrP^d^ accumulation to be detectable in tissues by conventional postmortem tests. However, PrP^d^ was detected in lambs culled at an earlier age that were fed different milk samples in this experiment. A similar finding was reported in an oral transmission study of chronic wasting disease (CWD) in deer: whilst PMCA allowed detection of PrP^sc^ in faeces and urine of CWD-affected deer, oral challenge with these excreta did not produce disease within 19 months based on conventional postmortem tests, even though PMCA and mouse bioassay demonstrated that these animals were subclinically infected [20]. Further studies would be required to confirm that the milk recipient was indeed infected, e.g. by inoculation of transgenic mice with tissues from the milk recipient.

Although the number of tested samples was very small, failure to detect PrP^sc^ by PMCA in two of three *ARX/ARQ* scrapie or BSE sheep-derived samples, but repeated detection in *VRQ/VRQ* scrapie sheep-derived samples, may suggest that shedding of PrP^sc^ (and possibly shedding of the infectious agent) is reduced in *ARX/ARQ* sheep or that other factors, such as strain (BSE or scrapie), genotype (*ARQ/ARQ* or *ARH/ARQ*) and sensitivity of the PMCA may contribute to the observed difference. Detection of PrP^d^ in the inguinal lymph node was suggestive of widespread PrP^d^ dissemination in the body but did not imply that the milk was infectious or harboured PrP^sc^ since it was also detected in the lymph node of the *ARQ/ARQ* BSE-affected sheep where the milk sample was PMCA-negative. Furthermore the BSE-affected ewe had reared two lambs (both *ARQ/ARQ*) for a period of 49 days when the milk sample that was tested for PrP^sc^ by PMCA was taken. Both were negative for BSE by postmortem tests on brain and LRS tissues when culled at 18 months of age (M Jeffrey, unpublished observation). Since experimental oral infection of sheep with this genotype usually results in PrP^d^ accumulation in LRS tissues before 18 months post infection [21,22], these data would indicate that the milk from this ewe was not infectious, supporting the PMCA-negative result.

Although feeding of milk from scrapie-affected *VRQ/VRQ* sheep was highly effective, scrapie-free sheep introduced to the milk recipient lambs from 72 days of age (to control for lateral transmission after mixing of sheep in Study 1), equally led to infection, with a median incubation period only slightly longer compared to milk-fed sheep. Thus, exposure to the infectious agent through other sources than the consumption of infected milk equally contributes to the risk of developing clinical disease. The longer incubation periods of these lateral transmission control sheep may be attributable to their older age when they were exposed to the scrapie environment, the lower infectious titre of the environmental source compared to milk or both. However, incubation periods should generally be interpreted with caution because they rely on defining the clinical end-stage, which is not always clear in sheep due to the variety of expressed clinical signs. For example, the lateral transmission control displayed clinical signs that were perceived as more severe and thus more advanced (disquilibrium, positional nystagmus and cataplexy-like episodes where the sheep was recumbent with motionless limbs) than the signs seen in milk-fed sheep where pruritus was the predominant sign.

The cause for the copper toxicity, which resulted in the premature cull of eight sheep in Studies 2 and 3, could not be identified. The copper levels in all analysed samples were below 10 mg/kg, which is the recommended dietary copper concentration for sheep without addition of counteracting molybdenum [23]. Copper intoxication has been reported in lambs housed in specific pathogen free (SPF) conditions and fed a diet with normal copper and molybdenum concentrations, which was attributed to the underdeveloped microflora in the rumen, particularly protozoa, which may inhibit copper absorption in the sheep by converting it in an insoluble form (e.g. CuS) or reducing the divalent copper ion to its less absorbable monovalent form [24]. Although sheep in the present study were not reared SPF, the accommodation was thoroughly cleaned and decontaminated with sodium hypochlorite before sheep were housed, and lambs were removed from their dams at birth, thus preventing transfer of protozoa via the dam. In addition, lambs were kept in groups of two for a considerably longer time in Studies 2 and 3 where copper intoxication occurred, thus reducing contact with individuals and limiting the contamination of drinking water, which may act as the main source of protozoal transmission [25]. Examination of rumen contents of the culled sheep would have been required to test this hypothesis, which was not done.

## Conclusions

Colostrum and milk from scrapie-affected ewes can transmit scrapie and results in a high transmission rate in sheep with a *VRQ/VRQ* genotype. Using PMCA PrP^sc^ was detectable in milk samples from selected ewes, which substantiates this method as a putative tool to study scrapie transmission *in vitro*.

## Methods

All procedures involving animals were approved by the United Kingdom (UK) Home Office under the Animals (Scientific Procedures) Act 1986.

Study 1 was initiated in 2005 to test the hypothesis that scrapie can be transmitted to sheep via milk. As there was no scientific evidence at the time that scrapie was transmissible via milk, the number of sheep required to test the hypothesis could potentially be very large. It was considered feasible to do a study with 59 scrapie-affected sheep conducted over four years, which would be sufficient to confirm transmission if 5% of sheep (with 95% confidence interval) excreted the scrapie agent and transmitted disease to a milk-fed lamb. The interim results of Study 1 [2] led to a modification of the project design, which was implemented in subsequent years, to address issues not known at the start of the study, such as the effect of potential lateral transmission, high somatic cell count and clinical status on the interpretation of the results (Study 2, started 2007), and to provide further information on infectivity of colostrum or milk (Study 3, started 2008).

### Study 1. Feeding milk from scrapie infected sheep to lambs with mixing of lambs

The methods have been described in detail previously [2]. Briefly, colostrum and milk (unless otherwise specified, colostrum and milk is subsequently referred to as milk) was collected from 12 *VRQ/VRQ* ewes naturally infected with scrapie either at clinical stage (eight) or pre-clinical stage (four sheep). The milk was frozen at below −20°C and fed subsequently to a total of 18 Cheviot *VRQ/VRQ* lambs from a flock free from classical scrapie [26]: milk from individual ewes was fed to newborn lambs in the same order it was collected (e.g. milk from day 1 was fed shortly after birth, followed by milk from day 2 etc.), without pooling of milk, i.e. milk from individual ewes was only fed to one particular set of two lambs or single lambs if the volume was insufficient to feed two lambs; milk was not pooled. Milk recipients of individual ewe’s milk were housed separately until all scrapie milk was consumed, after which they were mixed and fed milk replacer (Lamlac, Volac International Ltd., Royston, UK).

Five ‘lateral transmission control’ lambs (weaned lambs from the same scrapie-free flock) were mixed with the milk recipients aged 70–72 days (three lambs) and 112 days (two lambs) respectively to assess whether horizontal transmission between sheep occurs after mixing of milk recipients.

Ten *VRQ/VRQ* Cheviot lambs were housed in the same accommodation but separate pen as ‘building controls’ to control for environmental contamination. These lambs were kept with their dam until weaning age.

At approximately 9 months of age, the scrapie status was evaluated based on a biopsy of the rectal mucosa under local anaesthesia (mixture of Lidocaine 2.5% and Prilocaine 2.5%, EMLA cream 5%, AstraZeneca, London, UK) in live animals (15 scrapie milk recipients, all five lateral transmission controls, nine building controls) to check for the presence of PrP^d^ in RAMALT. For sheep that died of intercurrent diseases (three scrapie milk recipients, one building control) selected LRS tissue (distal ileum, mesenteric lymph node, spleen) and the brain were collected *post mortem* and examined for the presence of PrP^d^. Postmortem tests comprised the immunohistochemical examination of formalin-fixed and wax-embedded tissue sections of brain (obex) and LRS tissue, including RAMALT, using rat monoclonal antibody R145 as described previously [27]. A section of the obex was also routinely stained with haematoxylin-eosin (H&E) and examined for the presence of vacuolar changes. A fresh sample of the caudal medulla was additionally examined for the presence of PrP^res^ by Western immunoblot (Bio-Rad TeSeE Western blot, Bio-Rad Laboratories, Hemel Hempstead, UK) as described elsewhere [28].

All scrapie milk recipients and lateral transmission controls were culled upon development of definite clinical signs (clinical end-point). The clinical examination followed the same short examination protocol as used for scrapie-affected goats [29]. Building controls were culled after the last scrapie-affected sheep was culled. The clinical end-point was reached when animals displayed abnormalities in sensation (frequent pruritic behaviour and a positive scratch test or alopecia) or movement (ataxia or tremor).

### Study 2. Feeding milk from scrapie infected sheep to lambs without mixing of lambs

The milk from seven 18 month-old *VRQ/VRQ* ewes (Poll Dorset × Friesland) born in the VLA flock with a high incidence of scrapie [4], as mentioned in Study 1 above, was collected by hand and frozen at −80°C to be used in the following year. All ewes tested negative for MVV antibodies by Agar Gel Immunodiffusion Precipitin Test (AGIDT) using the Maeditect test kit (AHVLA Weybridge, Addlestone, UK) [30]. An aliquot of each weekly milk sample collected after the colostral period was sent for SCC determination (National Milk Records plc, Chippenham, UK). A routine bacteriological examination was carried out (by AHVLA Winchester, Hampshire, UK) if the SCC exceeded 10^6^ cells per ml. The scrapie status of each ewe was confirmed by immunohistochemical examination of a RAMALT biopsy collected prior to milking, at approximately one year of age, as described above. Assessment of clinical signs associated with scrapie in the lactating ewes was made by experienced animal husbandry staff familiar with the animals, who used a three point classification system to evaluate the clinical scrapie status. Mild signs included mild behaviour change, some pruritus with only minor fleece damage, minor muscle fasciculations; unequivocal signs included obvious behaviour change, pruritus with fleece damage but without skin lesions, tremor and some loss of weight or condition, and strong signs were ataxia, pruritus with minor skin damage and poor body condition.

Ewes were generally culled after development of at least unequivocal signs of scrapie, which was confirmed by a neurological examination prior to cull using the same methodology as described above. Disease was confirmed by histopathological (H&E) and immunohistochemical (antibody R145) examination of formalin-fixed and wax-embedded samples of the obex as described above as well as examination of a fresh sample of the caudal medulla by Western immunoblot (VLA Hybrid technique [31]).

The milk from the entire lactation was fed to a pair of *VRQ/VRQ* lambs born from ewes of the scrapie-free flock. The scrapie-free status of the ewes was confirmed by postmortem examination of the brain using immunohistochemistry (method as above) and ELISA (Bio-Rad TeSeE, Bio-Rad Laboratories) according to the manufacturer’s instructions [32]. In addition, the inguinal (mammary) lymph nodes and the mammary gland were examined immunohistochemically for the presence of PrP^d^ in lymphoid follicles.

The feeding protocol was as described for Study 1 (see above) but this time all pairs of lambs were kept in separate pens with separate equipment and entries to avoid lateral transmission. Prior to housing of sheep all pens were decontaminated with sodium hypochlorite (20% solution with 20,000 ppm available chlorine), which is effective against prions [33].

Milk replacer (Lamlac) was fed after all scrapie milk was consumed, followed by a diet of straw and concentrates at weaning age. Mixing of lambs only took place after PrP^d^ was detected in a RAMALT biopsy in at least one lamb of each pair. Biopsies were taken at 4.5 months of age and repeated two weeks later if the tissue was inadequate for a diagnosis. Five *VRQ/VRQ* Cheviot lambs were housed in a separate pen of the same accommodation (building controls) and kept on a similar diet as the scrapie milk recipients (colostrum from the scrapie-free dam, milk replacer, then straw/concentrates).

The procedures for culling of animals and the protocol for examination of tissue were identical to Study 1 (see above).

### Study 3. Feeding colostrum and milk from scrapie infected sheep to separate lambs

Milk from five 18–21 month-old *VRQ/VRQ* ewes from the VLA scrapie flock (one Poll Dorset, four Poll Dorset × Friesland) was collected. All ewes had no detectable antibodies against MVV by AGIDT. Identical to the previous study, milk was frozen and stored for a year and weekly samples were submitted for SCC. A RAMALT biopsy taken at 10 months of age in one and at 16 months of age in four ewes was examined immunohistochemically for the presence of PrP^d^ to confirm scrapie status. Assessment of the clinical status and disease confirmation by postmortem tests was made as described above, including immunohistochemical examination of inguinal lymph nodes and mammary gland.

The milk from each ewe was separated into colostrum (= lactation up to day 4) and milk (lactation from day 5). Recipient lambs were born from ewes derived – as before – from the classical scrapie-free flock; 15 of the dams were used for another project and no follow-up on their scrapie-free status was possible; the other four, dams of five lambs, were scrapie-negative on brain examination by ELISA (Bio-Rad TeSeE, Bio-Rad Laboratories). Scrapie colostrum and milk were fed to a pair of lambs so that one pair received scrapie colostrum only and one pair received scrapie milk only. Scrapie colostrum was fed within 10 minutes after birth whereas scrapie milk was fed within 23 hours after birth but not earlier than 9 hours after birth since these lambs first received colostrum from their scrapie-free dams. The subsequent procedure was identical to previous studies: feeding of scrapie colostrum and milk in the same order it was collected, without pooling of milk, feeding milk replacer after all colostrum or milk was consumed and strict separation of paired lambs until scrapie diagnosis was made from a RAMALT biopsy taken from 4 months of age. Building controls comprised four *VRQ/VRQ* Cheviot lambs housed in a separate pen of the same accommodation and kept on a similar diet as the scrapie milk recipients (colostrum from the scrapie-free dam, milk replacer, then straw/concentrates). Sheep were again housed in medium security accommodation that also contained sheep and building controls from the previous two studies. As before, pens were decontaminated with sodium hypochlorite prior to movement of lambs. The procedures for culling of animals and the protocol for examination of tissue were identical to the first two studies (see above).

### Application of PMCA to detect PrP^sc^ in milk

Milk samples were tested from six *VRQ/VRQ* scrapie-affected sheep that also provided milk for the transmission study to lambs (ewes from Study 3, see Table 2 and ewe 139/6 from Study 1, which fed one lamb culled at 105 days of age that was negative for scrapie by postmortem tests [2], see also “Additional file 1: summary”, which shows this dam and its milk recipient), with corresponding control samples from five healthy *VRQ/VRQ* sheep from the scrapie-free flock. Other milk samples were derived from one *ARQ/ARQ* sheep at 34 months post oral challenge with 5 g of BSE brainstem homogenate, which came from a BSE research flock [34], and two scrapie field cases (case 134/11: *ARQ/ARQ*, case 127/11: *ARH/ARQ*), with corresponding control samples from 11 healthy *ARQ/ARQ* sheep from the scrapie-free flock. With the exception of the samples that came from the two scrapie field cases that resembled colostrum, all tested samples were milk and not colostrum, i.e. they were collected on days after day 4 of lactation. Samples were collected at up to six different time points (depending on the length of lactation and availability of samples) and tested individually; in the *ARX/ARQ* TSE cases milk from each udder half was tested separately. Tables 3 and 4 list the animals with information on status of infection and lactation. Both scrapie field cases tested negative for antibodies against Maedi-Visna virus by AGIDT.

Inguinal lymph nodes and mammary gland of the sheep with BSE and the two scrapie field cases were examined by immunohistochemistry as described above.

Milk samples from ewes that also provided milk fed to lambs were collected weekly (volume 50 ml) and separated into whey, cream and cell fractions for further testing following a method described elsewhere [5] before being stored at −80°C. As whole milk was tested for comparison with the *in vivo* data, these fractions were thawed and recombined prior to further processing. All other milk samples were frozen at −80°C without being fractionated and thawed prior to testing. The methods used for the serial PMCA followed those described previously [8]. Briefly, whole or recombined milk samples were mixed for 1 minute with 9.5% (v/v) EDTA (V4231 Promega Corp, Madison, WI, USA), 0.28% (w/v) Sodium Deoxycholate (D6750 Sigma-Aldrich, St Louis, USA) and 0.28% (v/v) Igepal CA-630 (I3021 Sigma-Aldrich) and clarified by centrifugation at 16000g for 10 minutes at 10°C. Supernatants below the fat layer were diluted 1:10 in PMCA substrate and supplemented with polyadenylic acid (PA) (P9403 Sigma-Aldrich) at 100 μg/ml to enhance the efficiency of amplification [15]. Substrates were prepared from ovine brain tissue from a *VRQ/VRQ* sheep (PG1521/10) and an *ARQ/ARQ* sheep (PG0220/11) and were used for testing *VRQ/VRQ*-derived and *ARQ/ARX*-derived milk samples respectively; both brains were from sheep from the scrapie-free flock, which tested negative for TSE by ELISA (Bio-Rad TeSeE, Bio-Rad Laboratories). Diluted samples were subjected to four rounds (*VRQ/VRQ* samples) or 11 rounds (*ARQ/ARX* samples) of serial PMCA, diluting samples 1/3 in fresh substrate (same substrate with PA) between each round. Each round comprised 48 consecutive cycles of sonication (40 seconds at 250 W) and incubation (29 minutes 30 seconds). PMCA products were stored frozen at −20°C until analysed. Products were analysed by enzyme immunoassay (EIA) (IDEXX HerdChek BSE-Scrapie Antigen Test Kit, IDEXX Laboratories, Westbrook, USA) using a modified protocol. Briefly, PMCA products were diluted 1:5 in kit homogenisation buffer then 4:5 with kit plate diluent. The sample (100 μl) was applied to the capture plate for 180 minutes at room temperature (RT) (~20°C). Excess reagents were washed away (kit wash 1) and bound sample incubated with conditioning buffer for 10 minutes at RT. Following a further wash (kit wash 2) wells were incubated with the small ruminant anti-PrP horseradish peroxidase conjugate for 90 minutes at RT then followed by another wash (kit wash 2). Visualisation of bound PrP^Sc^ was achieved using 3,3′,5,5′-tetramethylbenzidine (TMB) substrate and measured at 450 nm using a reference filter at 620 nm (Perkin Elmer Envision 2104 multi-label reader). Amplification was determined by comparison with pre-amplification reference samples [15]. In each experiment negative control samples were included to monitor both *de novo* synthesis and putative contamination. The substrate only controls used in each experiment comprised of substrate spiked with extract from a control milk sample (derived from ewes N290 and K193 from the scrapie-free flock for the *VRQ/VRQ* sample and *ARQ/ARX* sample test runs respectively). Eight replicates were included in each experiment. Each sample was analysed four times. Following PMCA all samples with an absorbance of greater than 2 were counted as positive. We use PrP^sc^ to describe scrapie-associated prion protein from the PMCA experiments since they are not subjected to PK digestion prior to detection.

## Competing interests

The authors declare that they have no competing interests.

## Authors’ contributions

TK proposed the study with the help of SJB and HAS, wrote the manuscript and managed the project. SJB and HAS managed the projects supplying the sheep. TK performed the clinical examinations. Histopathological examinations were done by SJM, FJS, MMS and TK. AR carried out the PMCA studies with the help of LAT and LT, both of whom contributed to the writing of the manuscript. All authors read and approved the final manuscript.

## Supplementary Material

Additional file 1 **Summary.** This presentation gives an overview of the three studies by providing the identity of the scrapie-affected dams (milk donors), the identity of the corresponding lambs fed milk or colostrum (milk/colostrum recipients), the control lambs and the experimental outcome of the lambs (with the length of the arrows proportional to the age of the animal at cull). Click here for file

Additional file 2 **07-1092.** Female Cheviot *VRQ/VRQ* milk recipient, 07–1092, examined at 22 months of age. It displays a fine head tremor (note the fine movements of the ears), alopecia on the poll and a positive scratch test (nibbling of the wall when scratched) suggestive of scrapie at clinical end-point. Click here for file

Additional file 3 **07-1246.** Lateral transmission control (*VRQ/VRQ*) used in Study 1 where sheep were mixed after milk consumption and examined at 32 months of age (29 months after mixing with scrapie milk recipients). This sheep stands facing the camera with low head carriage and swaying of the head and body. Upon approach, when it attempts to run away, it collapses to the floor and remains in sternal recumbency. (In a separate event, which is not shown, it lies in lateral recumbency with twitching of the eyelids and vertical/rotatory nystagmus, which disappeared when the sheep was placed in an upright position.) Muscle strength and demeanour returned to normal after the episode: the sheep can be seen standing firmly with its back rubbing against the metal rack. Click here for file

Additional file 4 **07-1288.** Milk donor sheep (*VRQ/VRQ*) used for Study 3 where milk and colostrum was fed separately, which includes Poll Dorset × Friesland ewe 07–1288 (red spray mark 7) at 18 months of age and lactation day 15. Its gait and behaviour within the group and during milking is unremarkable. (A clinical examination including scratch testing at that time did not reveal any abnormalities). Three months later, which was 41 days after the end of lactation, this sheep was presented for cull as clinical suspect (strong signs of scrapie) with loss of bodily condition, pruritic behaviour and the display of a positive scratch test: scratching of the back elicits a nibbling response (‘nibble reflex’). It does not yet display evident gait abnormalities. Click here for file
