# Supplementary material for: Evidence of effective scrapie transmission via colostrum and milk in sheep
Source: BMC Vet Res. 2013 May 7;9:99. doi: 10.1186/1746-6148-9-99 (PMC3750761; doi:10.1186/1746-6148-9-99)

Study 1. Feeding milk from scrapie infected sheep to lambs with lambs housed together

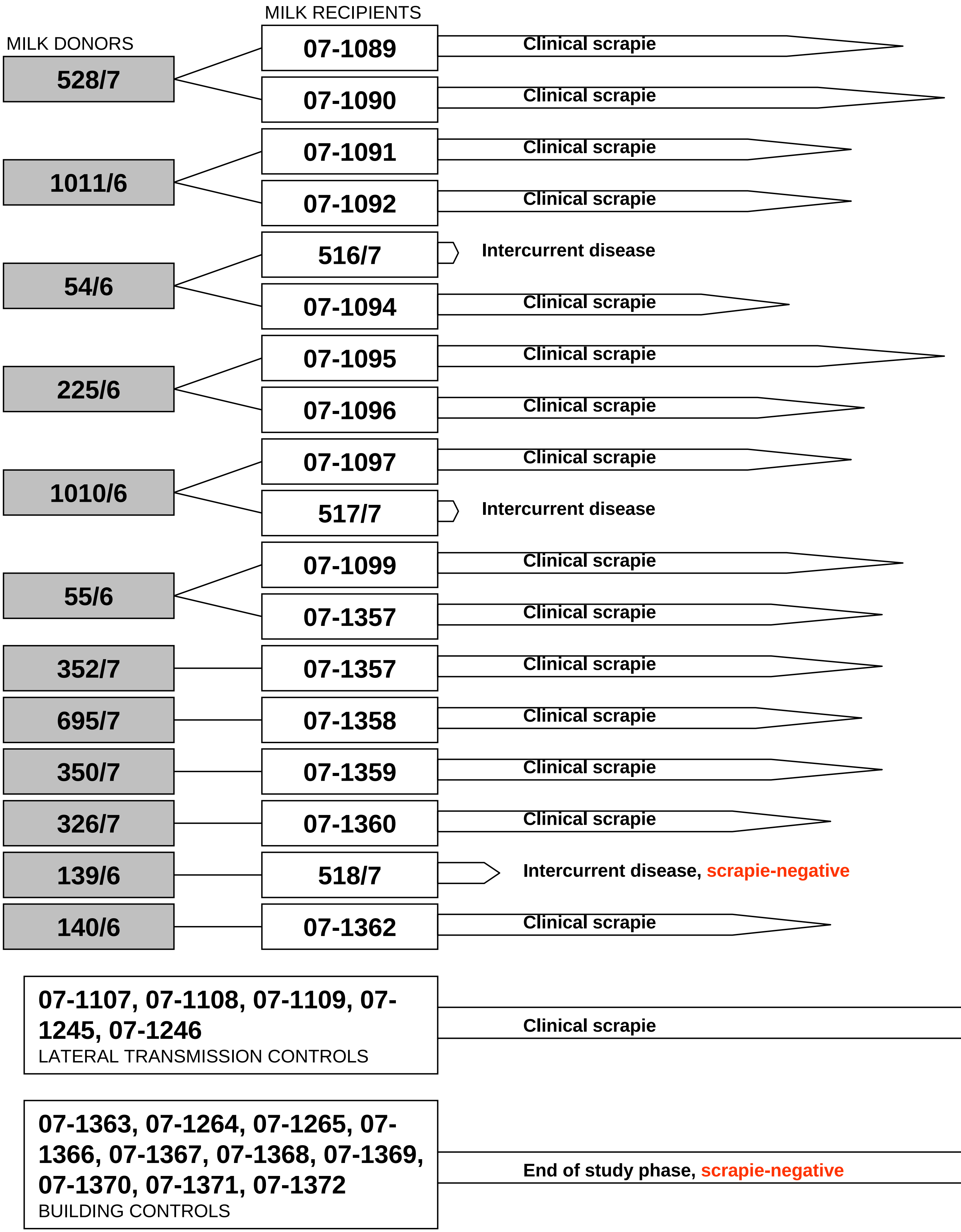

Study 2. Feeding milk from scrapie infected sheep to lambs without housing lambs together

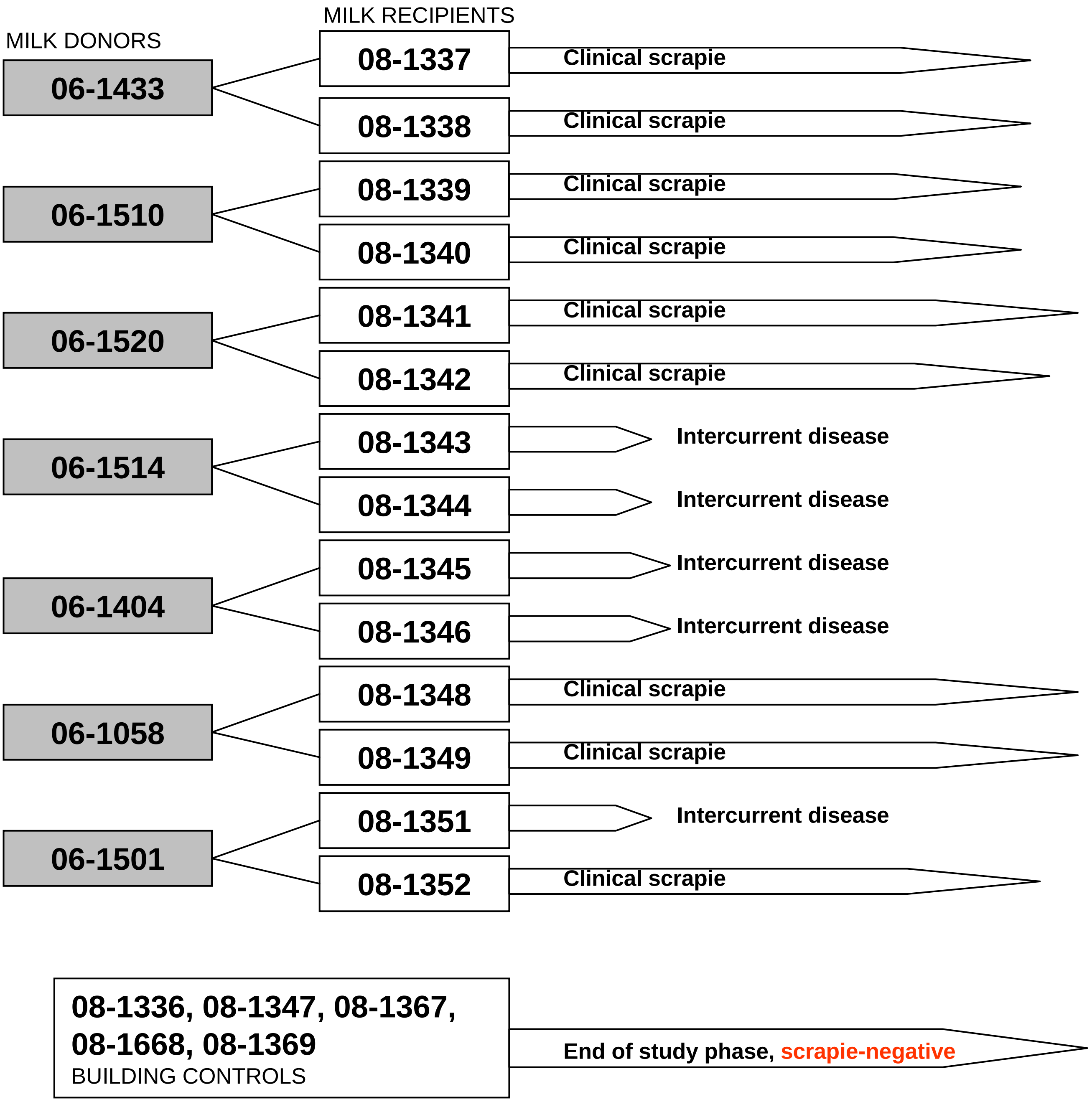

Study 3. Feeding colostrum and milk from scrapie infected sheep to separate lambs

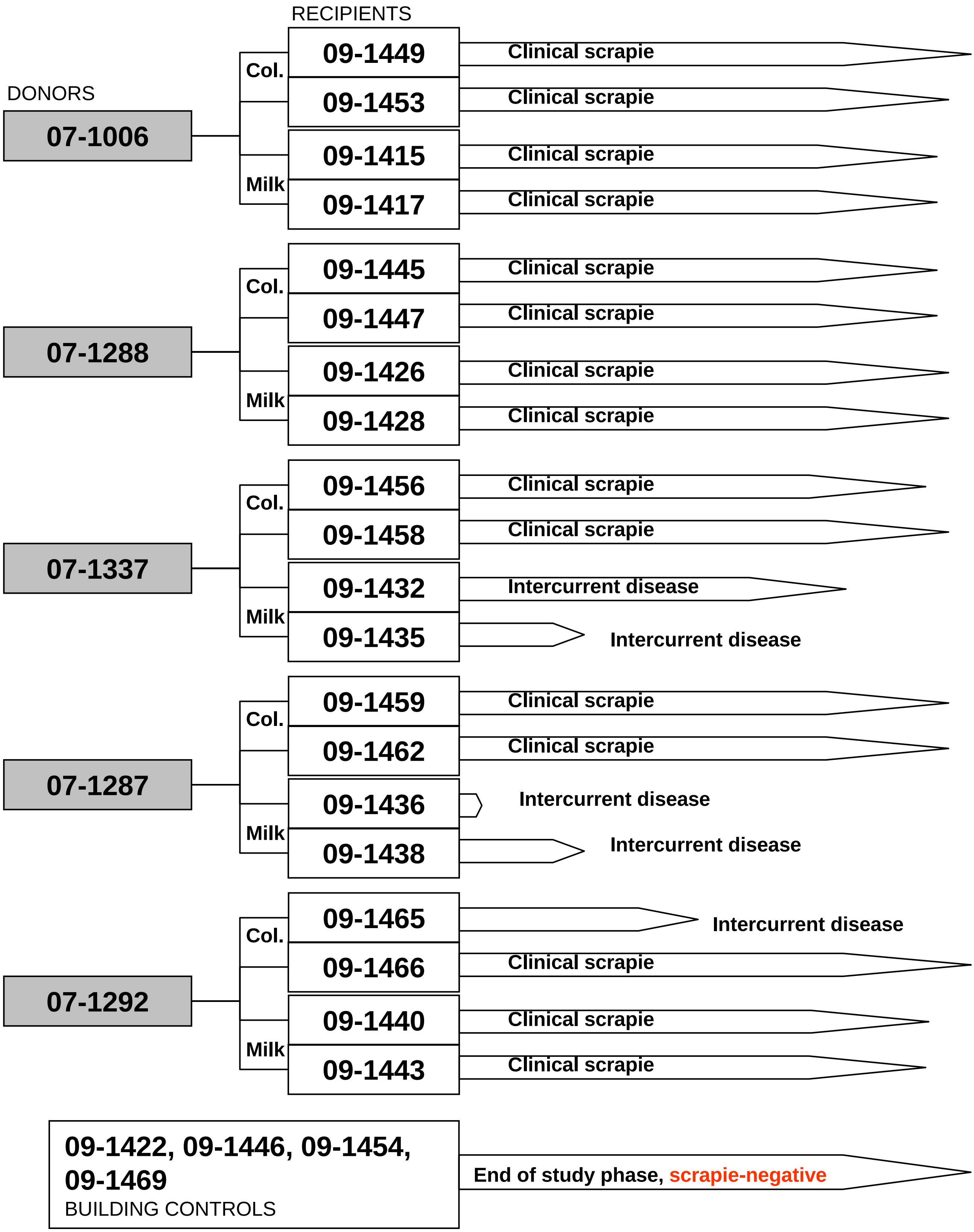

Supplement: Additional file 1 — Summary. This presentation gives an overview of the three studies by providing the identity of the scrapie-affected dams (milk donors), the identity of the corresponding lambs fed milk or colostrum (milk/colostrum recipients), the control lambs and the experimental outcome of the lambs (with the length of the arrows proportional to the age of the animal at cull). [file 1746-6148-9-99-S1.pdf]
